# Modeling the Invasion of the Large Hive Beetle, *Oplostomus* *fuligineus*, into North Africa and South Europe under a Changing Climate

**DOI:** 10.3390/insects12040275

**Published:** 2021-03-24

**Authors:** Hossam Abou-Shaara, Sara A. Alashaal, Eslam M. Hosni, Mohamed G. Nasser, Mohammad J. Ansari, Sulaiman Ali Alharbi

**Affiliations:** 1 Department of Plant Protection, Faculty of Agriculture, Damanhour University, Damanhour 22516, Egypt; hossam.farag@agr.dmu.edu.eg; 2 Entomology Department, Faculty of Science, Ain Shams University, Cairo 11566, Egypt; sara_alashaal@sci.asu.edu.eg (S.A.A.); mgnasser@sci.asu.edu.eg (M.G.N.); 3 Department of Botany, Hindu College Moradabad, MJP Rohilkhand University Bareilly, Bareilly 244001, India; mjavedansari@gmail.com; 4 Department of Botany & Microbiology, College of Science, King Saud University, Riyadh P.O. Box 2455, Saudi Arabia; sharbi@ksu.edu.sa

**Keywords:** climate change, invasion, pest, beekeeping, maxent, LHB

## Abstract

**Simple Summary:**

Large Hive Beetles (LHBs) are common pests of honeybee colonies, especially in the African continent. The ability of this pest to invade new regions in North Africa and Europe is highlighted in the present study using a species distribution modeling technique in current and future climate change scenarios in 2050 and 2070. In brief, this pest will be a new burden on the beekeeping sector outside Africa, and therefore the development of early monitoring strategies is recommended.

**Abstract:**

Some beetle species can attack honeybee colonies, causing severe damage to beekeeping. These pests include *Oplostomus fuligineus*, which is also known as the Large Hive Beetle (LHB). This beetle is native to Sub-Saharan Africa and has recently also been recorded in some parts of North Africa. It feeds mainly on young bee larvae and stored food within the colonies, causing severe damage to weak colonies. The present work sheds light on the current and future distribution (from 2050 to 2070) of this beetle in Africa and South Europe using species distribution modeling. Maxent was used to model the invasion of LHB. The Shared Socioeconomic Pathways (SSPs) 126 and 585 were used to model the future distribution of LHB. The Maxent models showed satisfactory results with a high Area Under Curve (AUC) value (0.85 ± 0.02). Furthermore, the True Skill Statistics (TSS) value was equal to 0.87. The current and future maps showed a high risk of invasion because of temperature variation in most of the parts of North Africa and South Europe. The maps also predicted the future invasion of LHB into other countries, mainly through southern Europe. These predictive risk maps will help quarantine authorities in highly relevant countries to prevent the expansion of this pest outside of its natural range.

## 1. Introduction

Invasive species are a major risk for current global biodiversity [1,2]. In addition to the ability of species to travel from one place to another through international transportation, global warming and climate change can accelerate a species’ expansion into new regions outside their natural habitat, with severe effects on the environment, economy, and sometimes human health [3]. The changing climate is having several effects on insect communities [4]. Insects have the ability to invade new regions influenced by increasing temperature through many ecological processes, including range extensions and phenological changes, as well as increased rates of population development, growth, migration, and over-wintering [5,6,7,8]. Distribution ranges can be greatly affected by such changes and can have a negative effect on human economy, especially with agricultural and medical pests [9,10]. Predicting the expansion of an insect pest becomes urgent for the prevention of economic losses, and so several modeling techniques have been developed to achieve this purpose (e.g.: Environmental niche modeling (ENM)) [7,11].

Environmental niche modeling depends on using species’ records against a panel of environmental parameters, including climatological data, to predict the current and future status of the species [12]. This technique becomes more soundly applicable through a number of studies including conservational and medical points of view. Also, it is considered to be a very effective tool for predicting species expansions and evaluating the effects of invasion [13,14,15,16,17]. Several modeling software using different mathematical algorithms have been developed in the last two decades for achieving this end, but Maxent (Maximum Entropy Model) is the most effective and accurate one [18,19,20]. The Maxent modeling is particularly used to predict the effect of climate change on several insect species including honeybee pests [21,22,23].

Apiculture faces several threats and problems [24,25]. For instance, a combination of negative factors threatens honeybee colonies (e.g., pests, intensive use of insecticide, global warming), thus increasing the occurrence of the colony collapse disorder (CCD) phenomena worldwide [26,27,28]. Today, the survival and productivity of honeybee colonies can be affected by several pests, including some beetle species. The major problem for honeybee colonies results from attacks by the Small Hive Beetle *Aethina tumida* Murray (Family: Nitidulidae), which can invade honeybee colonies to feed on stored pollen [29,30,31,32,33,34]. Another common beetle pest for beekeeping is the Large Hive Beetle (LHB).

Large Hive Beetles (LHBs) consist of two species of the genus *Oplostomus* that can attack honeybee colonies: *O. fuligineus* and *O. haroldi (Oplostomus* spp.: Coleoptera: Scarabaeidae) (Figure S1). These two species occur in Sub-Saharan Africa, but *O. fuligineus* is considered the most important honeybee pest. The two pests share approximately the same niche and biology in African countries. [35]. They prefer drier grazing land where dung is abundant and are generally absent from arid habitats such as the Kalahari Desert in Namibia and Botswana. They have three to four generations per year [25]. The immature stages develop in dung, while mating can take place in the beehives [35,36].

A heavy infestation with *O. fuligineus* can cause serious damage to honeybee colonies, especially to weak ones [36,37]. A severe infestation can exceed 700 beetles per colony and feeding can continue for more than thirty days [36]. The damage caused by these beetles includes feeding on young bee larvae and on stored food within the colonies [35]. They also have several morphological adaptations to protect themselves from bees, including their protective chitin exoskeleton [37]. The honeybees do not have any clear defensive mechanism against their attacks except for the use of propolis to seal the entrance to the colony and thereby to prevent the beetles from entering [38]. The distribution pattern of LHB is not clearly understood, especially in view of the beetle’s ability to fly and to invade new areas, migratory beekeeping, and the transportation of honeybee packages and equipment [30,31,39,40]. Adding to previous factors, the change in climatological conditions, mainly the increasing temperature, supports the spread of these insects outside their natural range [41,42]. Consequently, mapping the current and future distribution of LHB is urgently required, especially to limit its destructive effect on the apiculture economy. Connecting the huge number of heritage records of LHB to climatological information in order to model their environmental niche can help to achieve such a purpose [42].

The present study therefore aims to predict the current and potential future distribution of *O. fuligineus* (LHB) in Africa and South Europe using the Species Distribution Modeling (SDM) approach. The findings of this study are of great significance and provide an early alert for the protection of beekeeping in different geographical areas.

## 2. Materials and Methods

### 2.1. Occurrence Records

**Figure 1:**
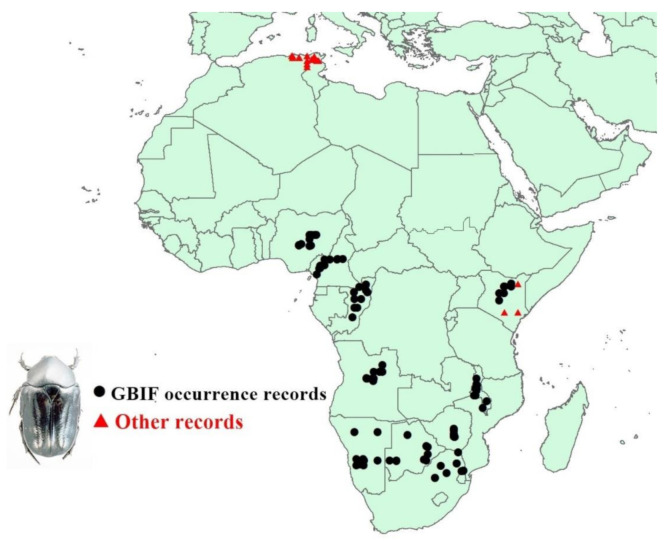
Occurrence record map showing the 118 occurrences, with the differentiation of Global Biological Information Facility (GBIF) data from other data.

*O. fuligineus* has been reported in Botswana, Namibia, Kenya, Zimbabwe, Nigeria, South Africa [35], Angola, Cameroon, Congo, Malawi (Global Biological Information Facility), and Tunisia, Algeria (online sources and personal communications). The distribution data of the large hive beetles (LHB) in these countries have been collected from different resources including the Global Biological Information Facility (GBIF.org (accessed on 20 October 2020) 2020: https://doi.org/10.15468/dl.3hew9t (accessed on 20 October 2020)) and published records. Duplicated and highly spatial uncertainty records were removed. A total of 118 occurrence records were used (Figure 1).

### 2.2. Current and Future Climatic Data

A total number of 19 bioclimatic variables was obtained (www.worldclim.org (accessed on 18 November 2020)), with a spatial resolution of approximately 5 km^2^. These data were originally derived from monthly temperature and rainfall values collected from forecast stations in 1950–2000. For current data, the bioclimatic layers were converted to ASCII format using ArcGIS v 10.3 and used to make a primary screening model to illustrate the most important variables in the distribution of LHB.

Multicollinearity and correlation among bioclimatic variables could prohibit the analysis of SDM [43]. For this reason, Pearson’s correlation was used to remove the autocorrelation among the 19 bioclimatic variables at (r^2^ ≥ |0.8|) [23,43]. This occurs through the function of SDM Tools in ArcGIS 10.3 (Universal tool; Explore climate data; Remove highly correlated variable) [23]. Only six temperature variables were selected and used to produce the final models. These biological variables were: Annual mean temperature (bio 1), Mean diurnal range (bio 2), Maximum temperature of warmest month (bio 5), Minimum temperature of coldest month (bio 6), Mean temperature of warmest quarter (bio 10), and Mean temperature of coldest quarter (bio 11), respectively.

For future data, parallel datasets of temperature variables were used from (www.worldclim.org (accessed on 18 November 2020)), covering the two periods 2050 and 2070 [44]. These data have been developed by climate centers to predict future temperature and consider two levels (126 and 585) of Shared Socioeconomic Pathways (SSPs). The SSPs are currently recommended as alternatives to Representative Concentration Pathways (RCPs) according to the Intergovernmental Panel on Climate Change (IPCC).

### 2.3. Species Distribution Modeling

Several software packages, such as CLIMEX, GARP, BIOCLIM and MaxEnt, have been used to suggest the potential distribution of invasive species under different climatic scenarios [45,46]. Nevertheless, the artificial intelligence of maximum entropy implemented in Maxent is considered to be the most frequently used package performing species distribution modeling using presence-only data [47,48]. Furthermore, Maxent has the ability to estimate the potential distribution even with a few occurrence records of the invasive pest [47,48].

Maxent v 3.4.1 was used to model the distribution of LHB in Africa and South Europe, using maximum entropy modeling [48]. In our models, 75% of the occurrence records were used for training, whereas 25% of the records were used for testing the model. The background points and the number of iterations were 10,000 and 1000, respectively. Moreover, the process was repeated in 10-fold cross-validation, which improved the model performance [49].

### 2.4. Model Performance

The contribution of each variable in the model was analyzed and the response curve of each variable was presented. Also, the Maxent outputs evaluated the model based on omission/commission rates of test and training points, the omission rate and predicted area of variables, the receiver operating characteristic (ROC), and jackknife tests of the variables. Values less than 0.5 indicated poor-fitting models while Area Under Curve (AUC) values of more than 0.75 indicated high-fitting of the models [50]. Furthermore, True Skill Statistics (TSS) was used, along with AUC, to estimate the accuracy of the predicted models [23]. The values of TSS range from −1 to 1, where positive values approaching 1 indicate a high relationship between the predictive model and the distribution, and negative values reflect a poor relationship [51].

## 3. Results

### 3.1. Predicted Current Potential Distribution

**Figure 2:**
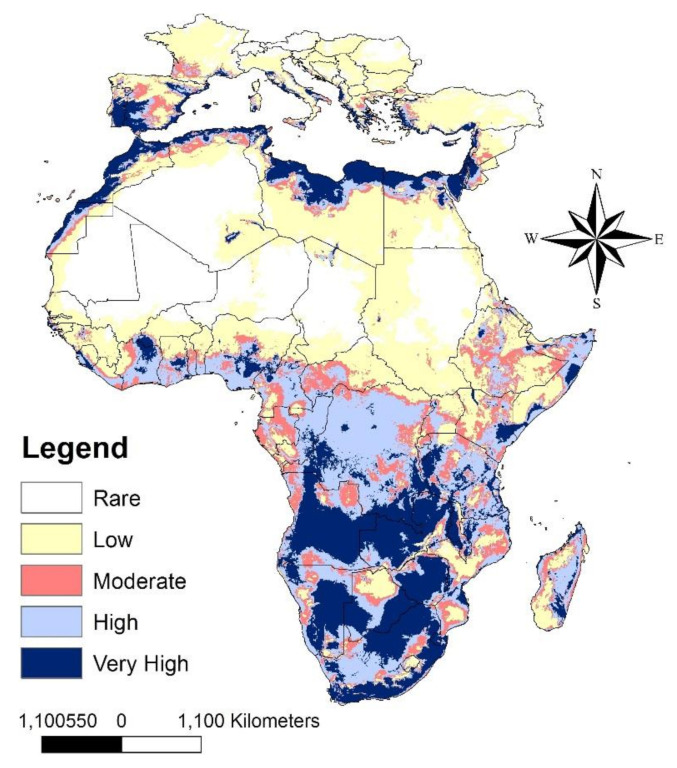
Current potential distribution of *Oplostomus fuligineus*.

Our predictive model appears to be good, with high AUC and TSS values equal to 0.85 ± 0.02 and 0.87, respectively. The predictive current map shows regions that are highly suitable for the distribution of LHB (Figure 2 and Figures S2). These regions cover the countries of Central and Southern Africa, in addition to coastal regions in Africa and some regions in South Europe. Deserts in Africa and northern parts in the European countries investigated showed less suitability for the occurrence of LHB.

### 3.2. Predicted Future Invasive Distribution (2050)

**Figure 3:**
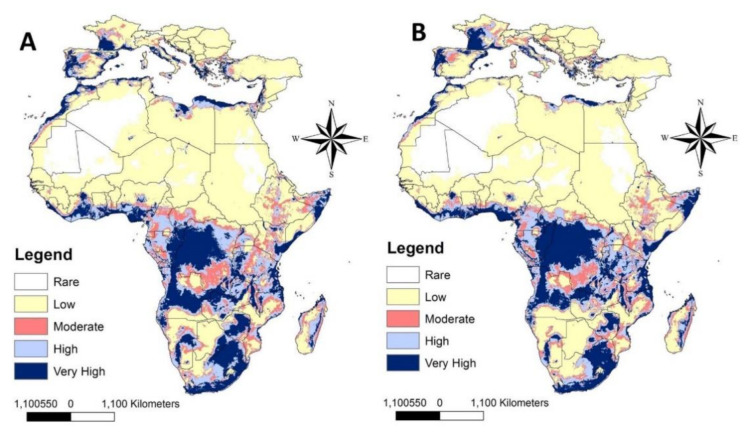
Predicted distribution of *Oplostomus fuligineus* in Africa and South Europe under future climate conditions in 2050; (**A**) for SSP 126, and (**B**) for SSP 585. SSP: Shared Socioeconomic Pathways.

Our predictive models show that at low SSPs (126), LHB could occupy new countries in Central and Southern Africa (Figure 3A and Figure S2). Countries located close to the Mediterranean Sea have a high potential to be invaded by LHB, including Spain, Italy, Greece, and Turkey, as well as coastal regions of Africa including Egypt, Libya, and Morocco. The less suitable regions for the occurrence of LHB included mainly the deserts of North Africa as well as some regions located in the northern parts of the European countries that were investigated. This situation slightly changed with the use of high SSPs (585) of future variables (Figure 3B). It is apparent that the less suitable regions in North Africa and South Europe are more than those on the map using the low limit of future variables. However, in both cases, LHB showed the ability to invade North African countries as well as Southern European countries (Figure 3A,B). It is expected that the routes of invasion will be mainly through coastal regions, across Africa to Morocco, as well as from Tunisia and Algeria to Egypt, the Levant, and then to Europe.

### 3.3. Predicted Future Invasive Distribution (2070)

**Figure 4:**
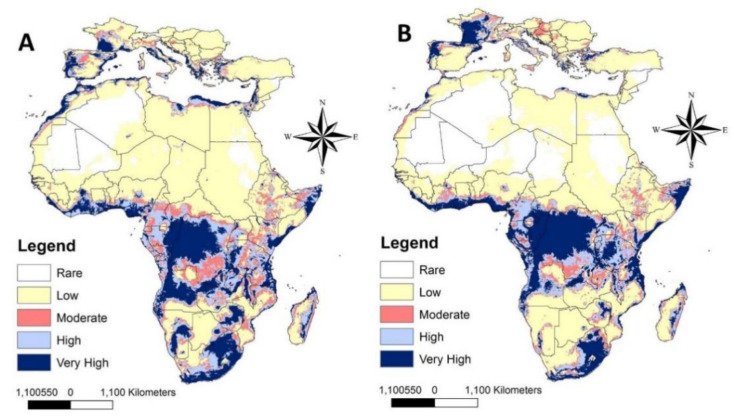
Predicted distribution of *Oplostomus fuligineus* in Africa and South Europe under future climate conditions in 2070; (**A**) for SSP 126, and (**B**) for SSP 585.

The predictive models of future potential distribution in 2070 based on low and high SSPs (126 and 585) showed similar expectations for the future invasion of LHB to North Africa and South Europe in 2050 (Figure 4A,B and S2). The differences between 2050 and 2070 are mainly in the low level of invasion by LHB to North Africa, except in the coastal regions in Egypt and Morocco. However, South European countries can be invaded by LHB in 2070 through the coastal regions in Africa, especially in Morocco, and from Egypt to Turkey and other European countries through the Levant region (Figure 4A,B).

### 3.4. Predicted Range Difference between Current and Future Distribution

**Figure 5:**
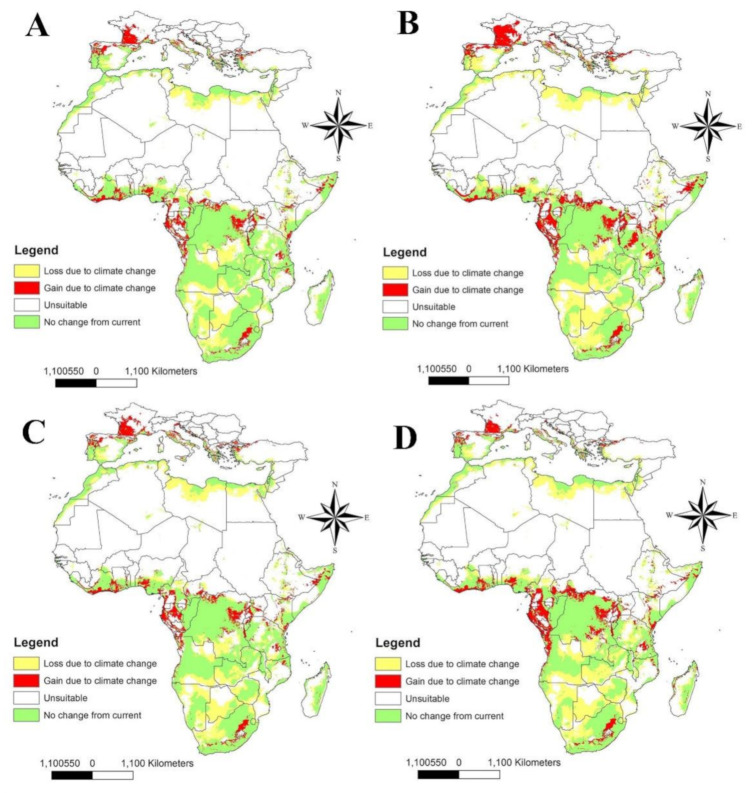
Calibration maps showing gain and loss in habitat suitability of LHB through the four future scenarios against current status: (**A**) 2050 for SSP 126; (**B**) 2050 for SSP 585; (**C**) 2070 for SSP 126, and (**D**) 2070 for SSP 585. LHB: Large Hive Beetle.

Calibration maps were used to evaluate the gain and loss through the overall suitability range of LHB, showing the differences between the current and future status of this pest (Figure 5). The 2050 SSP 126 is the most dangerous scenario for LHB expansion (Figure 5B), while 2070 SSP 585 shows less expansion (Figure 5D). All future scenarios indicate the suitability of habitats through northern Mediterranean countries for LHB invasion.

### 3.5. Model Performance and Variables Contribution

The Maxent model for LHB provided a high value of the Area Under Curve (AUC) equal to 0.85 (±0.02). The values of AUC in continuous species distribution modeling are high rather than discontinuous. Furthermore, the TSS value was high and equal to 0.87, which indicates excellent model performance. TSS values more than 0.5 are always acceptable.

**Figure 6:**
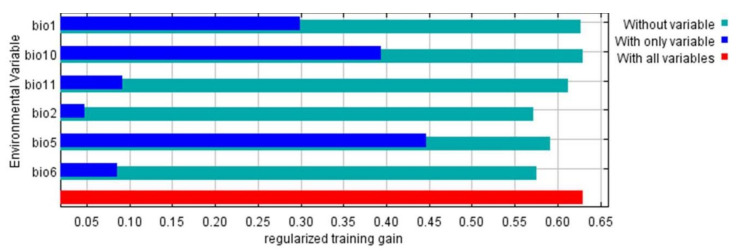
The jackknife test of the most important variables.

**Figure 7:**
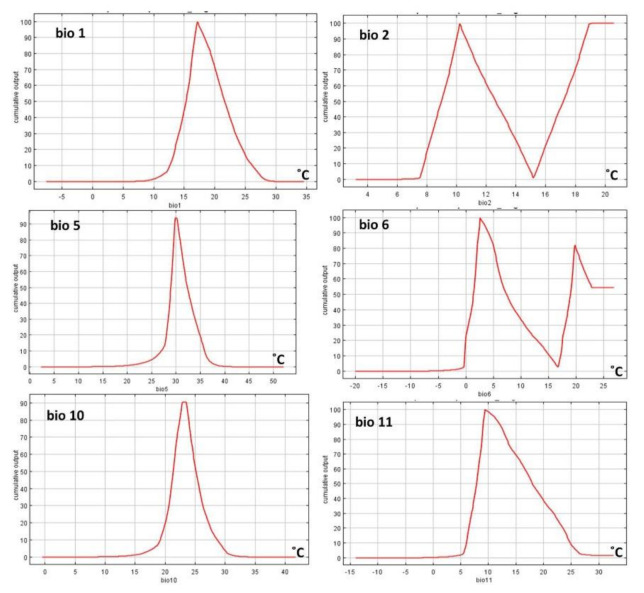
Response curves of temperature variables: annual mean temperature (bio1), and mean diurnal range (bio2), max temperature of warmest month (bio5), minimum temperature of coldest month (bio6), mean temperature of warmest quarter (bio10), and mean temperature of coldest quarter (bio11).

The relative contributions of the temperature variables to the Maxent model are illustrated in Figure 6. The highest contributed variable was bio5, followed by bio10, bio1, bio11, bio6, and bio2, respectively. In addition, the response curves showed that bio5 had a suitable value of 30 °C, while bio2 and bio11 had a value of about 10 °C, bio1 and bio10 about 20 °C, and bio6 about 5 °C (Figure 7).

## 4. Discussion

The health of honeybee populations is of global significance [52,53]. Honeybees are keystone pollinators in many natural and agricultural environments [54,55]. Such ecosystem services add to the honey production economy, which makes the honeybee one of the most important creatures in relation to human diet and medication [56,57]. So the study of honey bee pests and diseases is one of the most important fields of research, either from an economic or scientific point of view, but some of these pest species have been neglected or have been the subject of very few works dealing with their impact. One of these species is LHB [58,59,60,61,62]. The present work is considered to be the first study that contributes to an assessment of the impact of global warming on the expansion of LHB outside its natural range in Africa, using environmental niche modeling. The results of the current distribution clearly show the occurrence of LHB in countries of Central and Southern Africa. Indeed, these regions represent the original locations of LHB in Africa [35]. In addition to these regions, the map using the current temperature conditions shows the potential habitat suitability for LHB throughout North Africa and South Europe. The quarantine authorities of countries such as Egypt, Libya, Spain, and Italy should monitor these pests in order to prevent an expected invasion by these beetles shortly. Some other beetle pests were detected in the regions classified as highly suitable for LHB: for example, Small Hive Beetles were accidentally introduced into some countries without becoming widely established, including Egypt [31,63,64], Portugal [65,66,67], and Italy [68,69]. This highlights the potential invasion of LHB regions by new honeybee pests. In fact, LHB has not been detected so far in Europe and some North African countries. A study in Egypt as an example showed the presence of some nitidulid beetles that can attack honeybee colonies without any detection to Large Hive Beetles [32].

The forecast future distribution for LHB shows its high ability to invade new countries in Africa and South Europe in 2050 and 2070. This also suggests that LHB will become widely established in various countries. The maps show that specific regions will be highly suitable for the invasion of LHB, including coastal regions in North Africa as well as Mediterranean countries in Europe including Greece, Italy, Turkey, and Spain. Only the future map for 2070 using the high limit of SSP showed fewer occurrences than did other maps of LHB in North Africa and Europe, as the very high temperature will impose a considerable limitation on these beetles. The desert areas between Central and North Africa are also less suitable for the occurrence of LHB. This indicates the lesser suitability of high temperatures and dry conditions in these regions for the establishment of LHB. Such results are compatible with the absence of LHB from the Kalahari Desert and dry areas in Botswana [70]. The occurrence of these beetles throughout coastal regions is supported by previous observations in Kenya [37,71]. Generally, all the maps show a potential future expansion of LHB towards the north.

The prediction maps show that the expansion of LHB into new countries could occur through the coastal regions in Central Africa towards North Africa, and from North Africa to Europe (e.g., Morocco to Spain). This supports the suggested route through coastal regions for the future distribution of these beetles, as shown from model maps for current and future distribution. Many suggested scenarios for invasions into new areas through different pathways have been evaluated by entomologists, but all of them depend on human activities and commercial trade [72]. The trade of honeybees and apiculture tools could help in such a predicted spread of LHB to new regions, especially the trade in honeybee between South Africa and other European countries. Indeed, *Oplostomus fuligineus* has the potential to fly from one location to another following the emitted volatiles from honeybee colonies that could attract them [35,37]. LHB could thus invade new locations when climatic conditions are favorable to them. Red palm weevils of the genus *Rhynchophorus* provide a striking example of the effect of invasive species on the agricultural economy. They expanded from their native range in the Indian subcontinent and southeast Asia to the Middle East and Mediterranean region, destroying date production and the date industry throughout the invaded region [73]. The Maxent modeling was used to evaluate the risk of invasion by red palm weevils throughout the world, to help decision-makers to reduce the economic effect of such pests [74].

The terrible case of red palm weevil could be repeated with LHB, especially with the absence of a clear defensive mechanism against these pests in the European honeybee subspecies [30,75]. Furthermore, European honeybees do not tend to use propolis to seal the hive entrance as is done by African honeybees. The invasion of LHB into new environments could therefore cause serious damages to bee colonies if not managed by beekeepers. LHB can also attack paper wasp nests to feed on the brood as an alternative food [36,76]. These beetles even showed an ability to feed on certain fruits under laboratory conditions [36]. Such alternative foods could help in increasing the ability of these beetles to become established in new environments apart from apiaries. There is a good number of biological control agents to control bee pests, but such agents are likely to be absent in regions invaded by new pests [77]. Good monitoring protocols should therefore be followed to prevent the establishment of these beetles. Additionally, LHB has the ability to survive without any food or water for more than 30 days [35], and they could thus be transported passively with the trade in bee equipment from one location to another. Fortunately, there are some simple methods to control these beetles including the use of specific barriers in front of the hive entrances. Strong colonies can also defend themselves more effectively against these beetles than weak ones [35].

The maximum temperature of the warmest month made a greater contribution to the model that was produced than any other variable. The response curves show that a temperature from 5 to 30 °C is suitable for LHB, especially from 20 to 30 °C. This can be explained by the occurrence of LHB in the countries of Central and Southern Africa. In fact, the weather in these countries is warm, with temperatures of about 30 °C all through the year. It is known that temperature is an important factor for the development of beetles, including Small Hive Beetles [30,78]. The temperature increases in North Africa and South Europe due to changes in the climate in the near future explains the potential invasion of LHB into new countries in these regions.

Finally, the present work is a small step towards an understanding of the ecology of one of the neglected honeybee pests by providing an insight into the current and future status of these insects. The models that were produced were based on temperature variables to evaluate the impact of global warming, but adding more environmental and anthropogenic factors to the model could enhance the results, especially if used on a local scale.

## 5. Conclusions

This study shows the potential invasion of North Africa and South Europe in the near future by the Large Hive Beetle (LHB), and the possibility that it will become widely established. The study also presents the potential routes for the expansion of these beetles through Africa to Europe, considering coastal regions as being the most suitable for the future expansion of LHB, either from Central Africa to Morocco to Europe or from Egypt to the Levant to Europe. These beetles will be an additional burden on bee colonies and could lead to damage in apiaries and could negatively affect hive productivity. Countries highly at risk of being invaded by LHB should therefore take the necessary steps to prevent the invasion and establishment of LHB. This is the first study to model current and future expansion of LHB using temperature variables and Shared Socioeconomic Pathways. The results should encourage international co-operation between researchers to develop appropriate monitoring strategies as well as control methods for this pest.

## Data Availability

The data presented in this study are available in the article.

## Supplementary Materials

The following are available online at https://www.mdpi.com/2075-4450/12/4/275/s1, Figure S1: Large Hive Beetle (LHB) (*Oplostomus fuligineus)* invading a honeybee hive in north Tunisia. Figure S2: Habitat suitability variation in presence/absence classes for predictive current and future distribution of (LHB) and Summary of occurrence records map.
