# Supplementary material for: Modeling the Invasion of the Large Hive Beetle, Oplostomus fuligineus, into North Africa and South Europe under a Changing Climate
Source: Insects. 2021 Mar 24;12(4):275. doi: 10.3390/insects12040275 (PMC8063819; doi:10.3390/insects12040275)
Supplement: Supplementary file 1 [file insects-12-00275-s001.zip › Supplementary files.pdf]

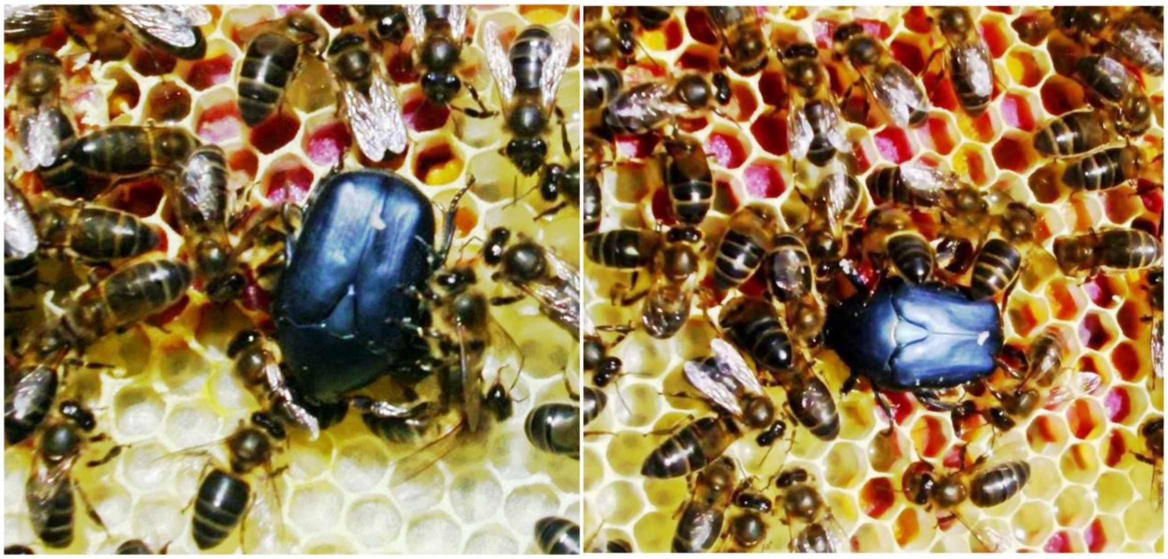

Figure S1: Large Hive Beetle (LHB) (*Oplostomus fuligineus)* invading honeybee hive in north Tunisia

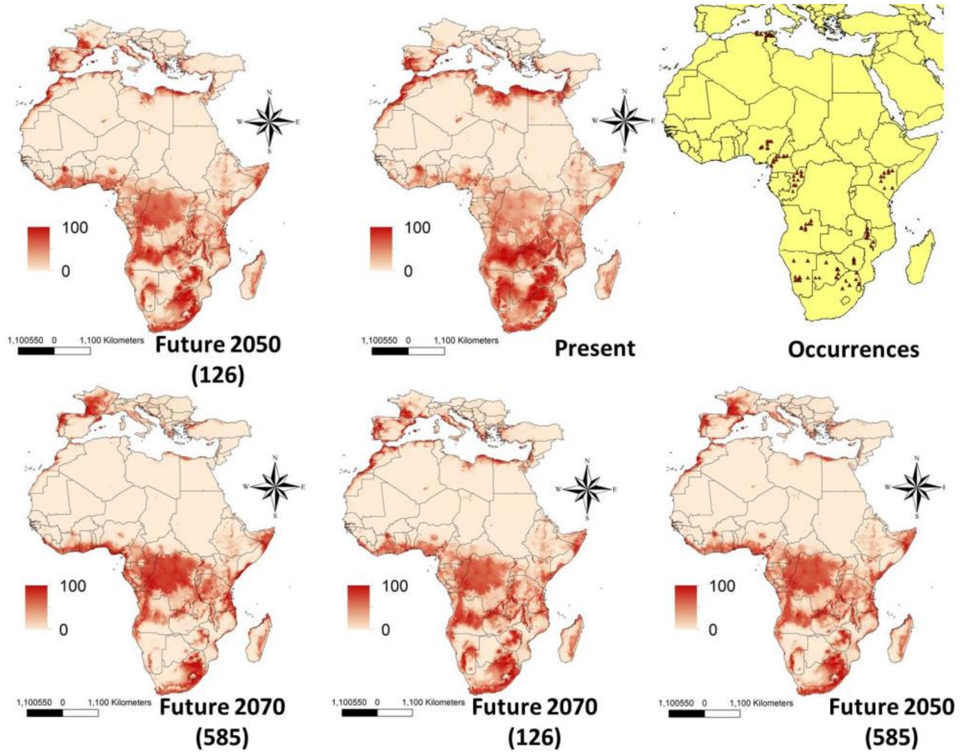

Figure S2: Habitat suitability variation in presence/absence classes for predictive current and future distribution of (LHB) and Summary of occurrence records map
